# Optical and spin-selective time-of-flight measurement of light-induced desorption of Rb from Fe$_3$O$_4$ surfaces

**DOI:** 10.1038/s41598-023-41937-1

**Published:** 2023-09-22

**Authors:** Kanta Asakawa, Naoki Tanabe, Taizo Kawauchi, Katsuyuki Fukutani, Atsushi Hatakeyama

**Affiliations:** 1 Department of Applied Physics, Tokyo University of Agriculture and Technology, Koganei, Tokyo 184-8588 Japan; 2 Institute of Industrial Science, The University of Tokyo, Meguro-Ku, Tokyo 153-8505 Japan; 3 Japan Atomic Energy Agency (JAEA), Tokai, Ibaraki 319-1195 Japan

**Keywords:** Atomic and molecular physics, Magnetic properties and materials, Surfaces, interfaces and thin films

## Abstract

Light-induced desorption of Rb atoms from a ferrimagnetic Fe$_3$O$_4$(001) surface was studied using a spin-selective optical method, which provides information on the spin polarization, velocity distribution, and amount of the desorbed atoms. The results showed that the intensity of the desorption of Rb from Fe$_3$O$_4$(001) induced by ultraviolet (UV) light irradiation was smaller than the detection limit at coverages lower than the threshold coverage at which the desorption rate began to increase. Moreover, the average magnetic quantum number of the desorbed atoms was smaller than that of electrons at the Fermi level of the Fe$_3$O$_4$(001) surface. These indicate that the light-induced desorption of Rb from an Fe$_3$O$_4$(001) surface occurs only in the high-coverage region in which the desorbing atoms are not in contact with the Fe$_3$O$_4$ surface, and that the desorption does not involve spin transfer.

## Introduction

Light-induced atom desorption (LIAD) from solid surfaces is attracting interest as a way to control the surface reaction or the vapor pressure of gaseous atoms in vacuum chambers^1–7^, which contributes to fundamental researches on catalytic activities^8^ and cold atom experiments^9^. Light-induced desorbed atoms can be detected by means of resonance enhanced multiphoton ionization spectroscopy^10^, laser-induced fluorescence^1^, light absorption^3,5^, Langmuir-Taylor detection^11^, two-photon laser-induced fluorescence^12^, and quadrupole mass spectrometry^13^. However, to our knowledge, the spin polarization of desorbed atoms has not been investigated. Adding spin sensitivity to the conventional methods of detecting LIAD will elucidate the spin dynamics of the adsorption and desorption processes. Alkali metal atoms often donate electrons to a surface on which they adsorb^14,15^. When they desorb from the surface, these atoms may receive electrons from the surface and become neutralized, an example of which is K atoms on a Cr$_2$O$_3$(0001) surface^10^. The spin of desorbed atoms depends on the spin of the electrons transferred from the surface. When alkali metal atoms adsorbed on a surface that have spin-polarized electronic states desorb as neutral atoms, the charge transfer between the surface and the adsorbate is likely to be spin-polarized. In such a case, the spin of the desorbed atoms will be spin-polarized. If the desorbed atoms are not spin-polarized, this would imply that either the desorbed atoms were not in contact with the spin-polarized surface or that spin relaxation occurred during desorption. An example of the former is the multilayer adsorption of alkali metal atoms, and an example of the latter is when desorption is driven by a thermal process that may have a long surface dwell time, during which the surface magnetism and the spin of the desorbing atoms may be disturbed^16–18^. In this study, we developed a method for detecting desorbed atoms with spin sensitivity. As a platform for demonstrating our method, we chose Rb atoms adsorbed on an Fe$_{3}$O$_{4}$ (001) surface.

Fe$_{3}$O$_{4}$ is a ferrimagnet that has been theoretically predicted to be a half-metal, in which the conduction electron is 100% spin-polarized^19,20^. Alkali metal atom adsorption on an Fe$_{3}$O$_{4}$ surface has been investigated theoretically by Yang et al.^15^, who reported that alkali metal atoms (Li, Na, K, Rb and Cs) donate electrons when they are adsorbed on an Fe$_{3}$O$_{4}$(111) surface. If the alkali metal atoms adsorbed on an Fe$_3$O$_4$ surface desorb as neutral atoms as a result of light irradiation, they must receive spin-polarized electrons from the surface. In such a case, they are likely to be spin-polarized, because surface electrons are spin-polarized. Our results show that the coverage dependence of the LIAD intensity exhibited a threshold coverage at which the LIAD intensity started to increase. Furthermore, no significant spin polarization of desorbed atoms was detected under the current experimental conditions. This implies that LIAD of Rb from Fe$_{3}$O$_{4}$(001) occurs only in the high-coverage region and that the degree of spin transfer that desorbing atoms receive from the surface is smaller than the detection limit.

## Experiment

A $10\times 10$ mm$^2$ MgO(001) substrate for an Fe$_3$O$_4$ film was cleaned by annealing at 973 K for 20 min under an oxygen atmosphere of $3\times 10^{-4}$ Pa before deposition. A 20-nm-thick Fe$_{3}$O$_{4}$ thin film was grown on an MgO(001) substrate by depositing Fe at a deposition rate of 0.44 nm/min for 23 min under an oxygen atmosphere of $3\times 10^{-4}$ Pa, using electron beam evaporation in an ultra-high-vacuum chamber with a base pressure lower than $1\times 10^{-8}$ Pa. The surface crystallinity was checked by reflection high-energy electron diffraction (RHEED). The RHEED pattern after deposition showed good agreement with that of the Fe$_3$O$_4$(001) surface^21^ to ensure that an epitaxial Fe$_3$O$_4$ (001) film was obtained. The sample was magnetized in air by applying a magnetic field of 1.0 T along the $\langle110\rangle$ easy magnetization direction^22^ using an electromagnet. Although the surface became contaminated and oxidized by air exposure, we consider that the chemical composition inside the Fe$_3$O$_4$ film was not affected because the conversion-electron Mössbauer spectra of Fe$_3$O$_4$ taken in air in Refs.^23,24^ showed good stoichiometry. The sample was then loaded into the ultra-high-vacuum measurement chamber.

**Figure 1:**
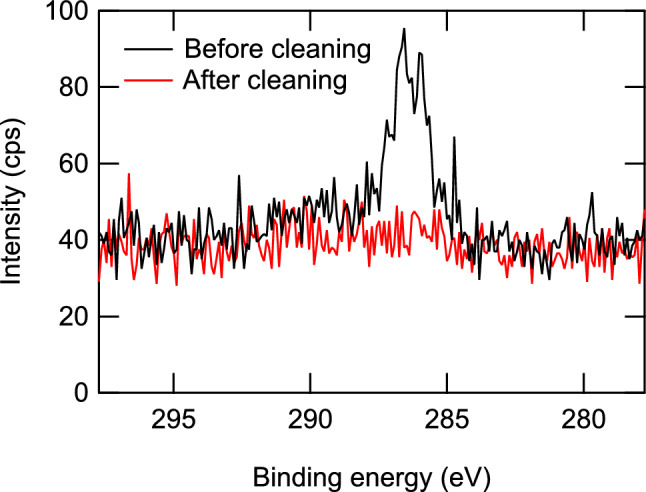
XPS spectra of the Fe$_3$O$_4$(100) surface around the C 1s-derived peak taken before and after the cleaning.

Before the measurement, the sample was cleaned by annealing it at 503 K in an oxygen atmosphere of $\sim 1.5\times 10^{-4}$ Pa for 19 h. Vescovo et al.^25^ reported that this method can recover the electronic structure and the stoichiometry of Fe$_{3}$O$_{4}$ (001) surfaces even after air exposure and that the surface obtained by this method exhibited an electronic structure closely resembling that obtained by cleaving the bulk single crystal and a clear Verwey transition, which ensure the stoichiometry of the surface. We checked the chemical composition of the surface using X-ray photoelectron spectroscopy (XPS). The result is shown in Fig. 1. The XPS spectrum taken before the cleaning exhibited an impurity-derived C 1s peak. No other impurity-derived peaks were found. After the cleaning, the C 1s peak became smaller than the uncertainty, which was 0.089% of the intensity of the Fe 2p-derived peak. From this, we estimated the density of the C atoms of the surface after cleaning. The probing depth *d* of XPS in nanometers is approximated as^26^

1 $$\begin{aligned} d=\frac{641}{E^2}+0.096\times \sqrt{E}, \end{aligned}$$

where *E* is the electron energy above the Fermi level in eV. For the Fe 2p-derived peak, we obtained $d=2.7$ nm. From the lattice constant of Fe$_{3}$O$_{4}$^27^ the areal density of Fe atoms within the probing depth was estimated to be $1.06\times 10^{16}$ cm$^{-2}$. By using the atomic sensitivity factors of 0.296 and 2.957 for C 1s and Fe 2p, respectively^28^, the areal density of C atoms was estimated to be smaller than $9.4\times 10^{13}$ cm$^{-3}$, which corresponds to 0.17 ML, where 1 ML is defined as the density of Fe atoms in the topmost layer^20^. This estimation ensured that a clean Fe$_{3}$O$_{4}$(001) surface was obtained by the present cleaning procedure.

**Figure 2:**
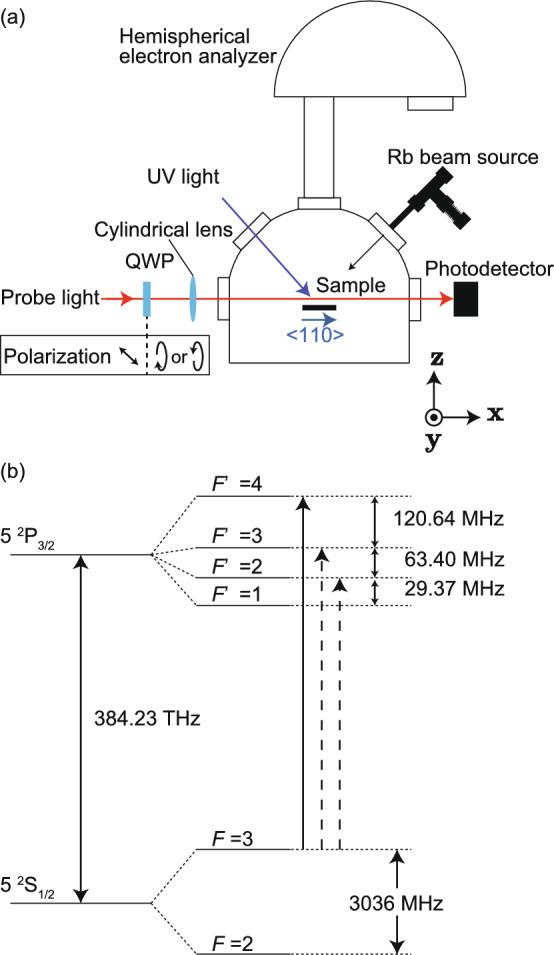
(**a**) Schematic illustration of the experimental setup and (**b**) energy diagram of the $^{85}$Rb $D_2$ transition lines from the $F=3$ ground state. QWP denotes the quarter-wave plate.

Figure 2a shows a schematic diagram of the experimental apparatus, which consisted of a measurement chamber with a base pressure of $\sim 2\times 10^{-7}$ Pa and an optical system for the probe light. The measurement chamber was made of permalloy to shield against the geomagnetic field. The stray field of the sample was so small that the influence of Larmor precession could be neglected in the spin measurement. The measurement chamber was equipped with a multi-channel effusive atomic beam source directed at the sample, viewports for the probe light and UV light, an Al K$\alpha$ X-ray source (not shown in Fig. 2a), and a hemispherical electron analyzer. The full width at half maximum of the cross-section of the Rb beam generated by the effusive atomic beam source was 8.2 mm at the position of the sample. Details of the effusive atomic beam source were described in Ref.^18^. The flux intensity was estimated to be $10^{12}$–$10^{13}$ atoms per second based on the designed value and the fluorescence induced by the probe light.

The probe light and pulsed UV light were introduced into the chamber through the viewports. The UV light was generated by the third harmonic of an Nd:YAG laser. The wavelength, pulse width, repetition rate, $1/\text{e}^2$ half width, and fluence of the third harmonic of the Nd:YAG laser were 355 nm, 5–10 ns, 10 Hz, 7.0 mm, and 81.2 mJ/cm$^2$, respectively. Figure 2b shows an energy diagram of the $^{85}$Rb $D_2$ transition lines. The probe light was generated by an external-cavity diode laser whose frequency was tuned to the $F = 3 \rightarrow F' = 4$ transition frequency of the $^{85}$Rb $D_2$ transition line by a frequency servo unit that utilized polarization spectroscopy^29,30^, as shown by the solid arrow in Fig. 2b. The line width of the probe light was estimated to be smaller than 1 MHz. Here, *F* and $F'$ are the total angular momentum of atoms in the $5^2\text{S}_{1/2}$ and $5^2\text{P}_{3/2}$ states, respectively. This probe light frequency enabled detection of $^{85}$Rb atoms whose velocity in the probe light direction was $0.0\pm 2.4$ m/s, as determined by the natural linewidth of the Rb $D_2$ transition line (6.06 MHz)^31^. A quarter-wave plate (QWP) mounted on a piezo-driven rotation mount made the probe light circularly polarized and allowed for variation in the helicity thereof. The intensity of the probe light was 8 $\upmu$W, which was sufficiently weak to prevent optical pumping. The probe light was oriented parallel to the $\langle110\rangle$ direction of the sample, i.e. the direction in which the magnetizing field was applied. The distance between the sample surface and the probe light was 1.0 mm. The light was condensed in the direction perpendicular to the surface by a cylindrical lens; its $1/\text{e}^2$ half widths in the surface-normal and surface-parallel directions were 0.40 and 1.0 mm, respectively, at the position of the sample. The intensity of the probe light passing through the chamber was measured using a high-speed photodetector, and the signal from the photodetector was processed using an oscilloscope. Desorbed atoms absorbed the probe light, as indicated by a dip in the time spectrum of the transmitted probe light intensity. Because the length of a desorbed atom cloud in the probe light direction is considered to be similar to the sample length, which was 10 mm, the detection limit of the density of atoms that resonate with the probe light is typically $\sim 10^8$ cm$^{-3}$ under the conditions presented. If we assume that the mean velocity of a desorbed atom is $10^4$ cm/s and that the width of the time-of-flight distribution is $10^{-5}$ s, we can approximately estimate the minimum detectable number of atoms that desorb by a single UV laser shot and resonate with the probe light to be 10$^7$. The probe light was also absorbed by the Rb atoms in the incident beam and the Rb atoms that were scattered on the surface. However, because the density of these atoms was much lower than that of the atoms desorbed by the pulsed UV light, the absorption of the probe light by these atoms could be neglected in the present condition. To estimate the spin polarization of the desorbed atoms, the helicity of the probe laser was switched every 20 shots. Rb beam irradiation and UV light irradiation were initiated simultaneously, and the sample was irradiated continuously with the Rb beam during the measurements.

## Results and discussion

**Figure 3:**
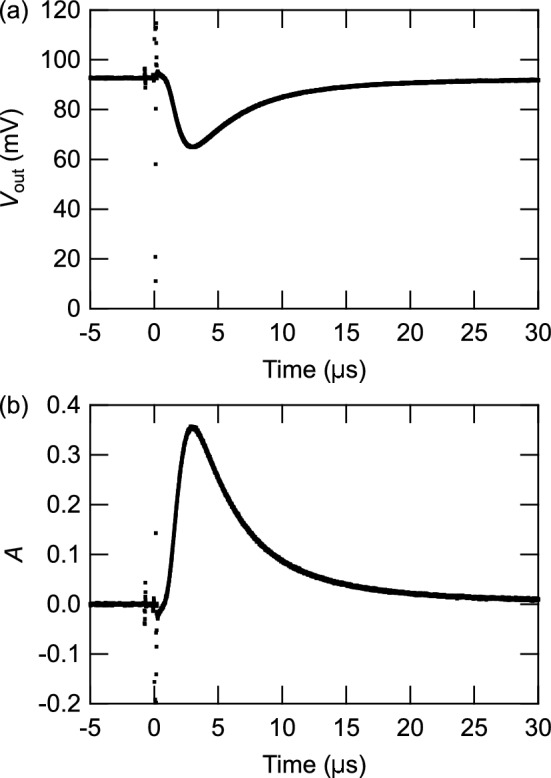
Time dependence of (**a**) the photodetector output signal and (**b**) the absorbance taken at $9.72\times 10^3$s after initiating the Rb beam irradiation. The noise at the time origin is due to the irradiation of the pulsed UV laser.

**Figure 4:**
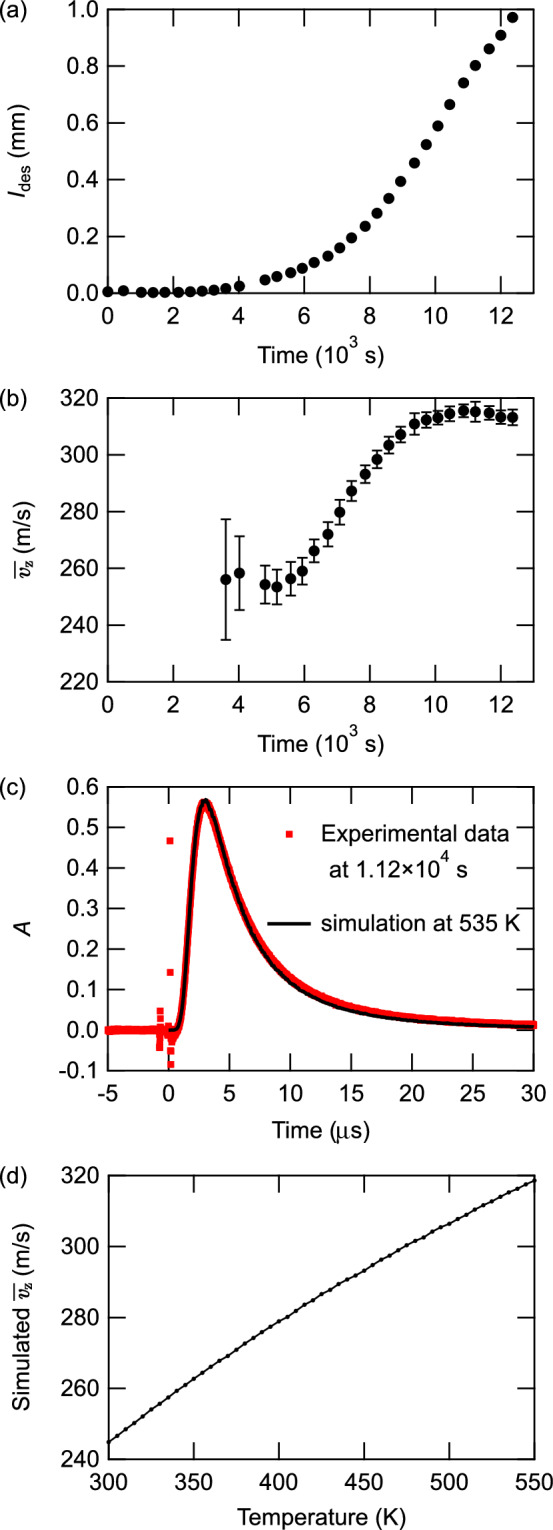
(**a**) Time evolution of $I_{\text{des}}$ and (**b**) mean velocity $\overline{v_{z}}$, (**c**) the simulated time spectrum of absorbance at 535 K compared with the experimental data taken $1.12\times 10^4$ s after initiating the Rb beam irradiation and (**d**) the simulated mean velocity of the desorbed atoms in the surface-normal direction as a function of temperature.

Figure 3a shows the time spectrum of the photodetector output $V_\mathrm{out}$ averaged over 2000 shots taken at $9.72\times 10^3$ s after the start of Rb beam irradiation. The noise around the time origin is due to the UV light. A dip was observed a few $\upmu$s after UV light irradiation, which is considered to have been due to absorption of the probe light by desorbed Rb atoms. Figure 3b shows the absorbance as a function of time, which is defined by $A(t)=\ln (V_{0}/V_\mathrm{out}(t))$. Here, $V_0$ is the baseline of the photodetector output voltage obtained by averaging the signal in the region $-87.5 \text{ }\upmu \text {s}<t<-2.5\text{ }\upmu \text {s}$.

To estimate the dependence of LIAD intensity on the UV and Rb beam exposure time, we introduced a quantity $I_\text{des}$, which was considered to be proportional to the number of desorbed atoms resonant with the probe light, as follows:

2 $$\begin{aligned} I_\text{des}= & {} \int _{t_1}^{t_2}A(t) v_{z} (t)dt\nonumber \\= & {} \int _{t_1}^{t_2}A(t)\cdot \frac{ l}{t} \text{d}t, \end{aligned}$$

where $v_{z}$ is the velocity component along the surface-normal direction and *l* is the distance between the sample and the probe light, which was $1.0\times 10^{-3}$ m. The integral interval is given by, $t_1=1.3$ $\upmu$s and $t_2=42$ $\upmu$s, which covers the whole peak and excludes the UV-laser-derived noise around the time origin. It should be noted that *A*(*t*) measures the density of resonant atoms rather than the flux, which means that the flux is proportional to $A(t) v_{z} (t)$^12^. Figure 4a shows the time evolution of $I_\text{des}$. The time origin is the start time of the Rb beam and UV light irradiation. It is considered that the coverage of Rb increases with time. However, since the adsorption probability and the efficiency of LIAD depend on the coverage, the coverage may not be proportional to time. Therefore, here we plot the result as a function of time. For each data point, $I_\text{des}$ was calculated from the spectra averaged over 2000 shots of UV laser pulses. Before $3.24\times 10^3$ s, the time spectra of the photodetector output signal showed no significant dip and, therefore, $I_\text{des}$ was below the detection limit. From $3.24\times 10^3$ s to the end of the measurement, $I_\text{des}$ increased without saturation. This implies the existence of a threshold coverage of Rb at which the LIAD intensity begins to increase, meaning that the desorption cross-section is smaller below the threshold coverage.

The mean velocity $\overline{v_{z}}$ of the desorbed atoms in the perpendicular direction was estimated from the time spectra using the following equation:

3 $$\begin{aligned} \overline{v_{z}}= & {} \frac{\int _{t_1}^{t_2}A(t) v_{z}^2(t)dt}{\int _{t_1}^{t_2}A(t)v_{z}(t)dt}\nonumber \\= & {} \frac{\int _{t_1}^{t_2}A(t)\left( \frac{l}{t}\right) ^2dt}{I_\text{des}}. \end{aligned}$$

Figure 4b shows the time evolution of $\overline{v_{z}}$. The absorbance was not large enough to obtain reliable results in the region between 0 and $3.60\times 10^3$ s. At $3.60\times 10^3$ s, $\overline{v_{z}}$ was $256\pm 21$ m/s. The mean velocity continued to increase over time, before plateauing at $\sim 315\pm 2$ m/s at $\sim 1.08\times 10^4$ s. To investigate whether the desorption mechanism is thermal or nonthermal, we simulated the time spectrum by a Monte-Carlo method. The simulation was performed by calculating the contributions of $4\times 10^8$ desorbed atoms with various positions and velocities to the time spectra of absorbance. We assumed the velocity distribution of desorbed atoms to be described by the Maxwell-Boltzmann distribution and that the angular distribution obeys Knudsen’s cosine law. Based on these assumptions, the distribution of atoms with velocity $\varvec{v}$ can be written as:

4 $$\begin{aligned} f(\varvec{v})\propto & {} \exp \left( -\frac{m|\varvec{v}|^2}{2k_\text{B}T}\right) \times v_{z} \nonumber \\= & {} \exp \left( -\frac{m(v_{x}^2+v_{y}^2+v_{z}^2)}{2k_\text{B}T}\right) \times v_{z} \end{aligned}$$

where *m* is the mass of an $^{85}$Rb atom, $k_\text{B}$ is the Boltzmann constant, and $v_{x}$ and $v_{y}$ are the velocity components along the *x* and *y* axes, respectively, which are shown in Fig. 2a. That the absorbance of the probe light measures the density of Rb atoms rather than the flux was taken into consideration. We also took into account the spatial intensity distribution of the probe light and the velocity selectivity of the transition by the probe light due to the Doppler shift. The simulated time spectra of absorbance were in good agreement with the experimental data, an example of which is shown in Fig. 4c. The simulated $\overline{v_{z}}$ is plotted against the temperature of the desorbed atoms in Fig. 4d. The simulation results imply that the range of the experimentally measured $\overline{v_{z}}$ values (253–316 m/s) corresponded to temperatures between 325 and 535 K, which were 21–231 K higher than the room temperature of 304 K. These values were consistent with the temperature of Xe atoms thermally desorbed from a bulk Au surface cooled to 23 K by an irradiating pulsed laser at a fluence of 50 mJ/cm$^2$ in Ref.^13^, which was $\sim 300$ K at maximum. The time evolution of $\overline{v_{z}}$ can be explained by assuming that the desorption is driven by a thermal process. The temperature of the surface increased with pulsed UV light irradiation. At low coverage, the desorbable adsorbed Rb atoms were depleted before the surface temperature peaked. However, at high coverage, a significant number of desorbable Rb atoms remained on the surface. As the desorption rate was high at high temperatures, the total velocity distribution of the desorbed atoms was dominated by the atoms that desorbed at high surface temperature. This explains the increase of $\overline{v_{z}}$ with increasing coverage. When the coverage was so high that a significant number of desorbable atoms remained on the surface when the surface temperature reached the peak, the atoms that desorbed around the peak temperature dominated the velocity distribution, and further increasing the coverage did not increase $\overline{v_{z}}$. This explains the saturating behavior of $\overline{v_{z}}$ at high coverages.

**Figure 5:**
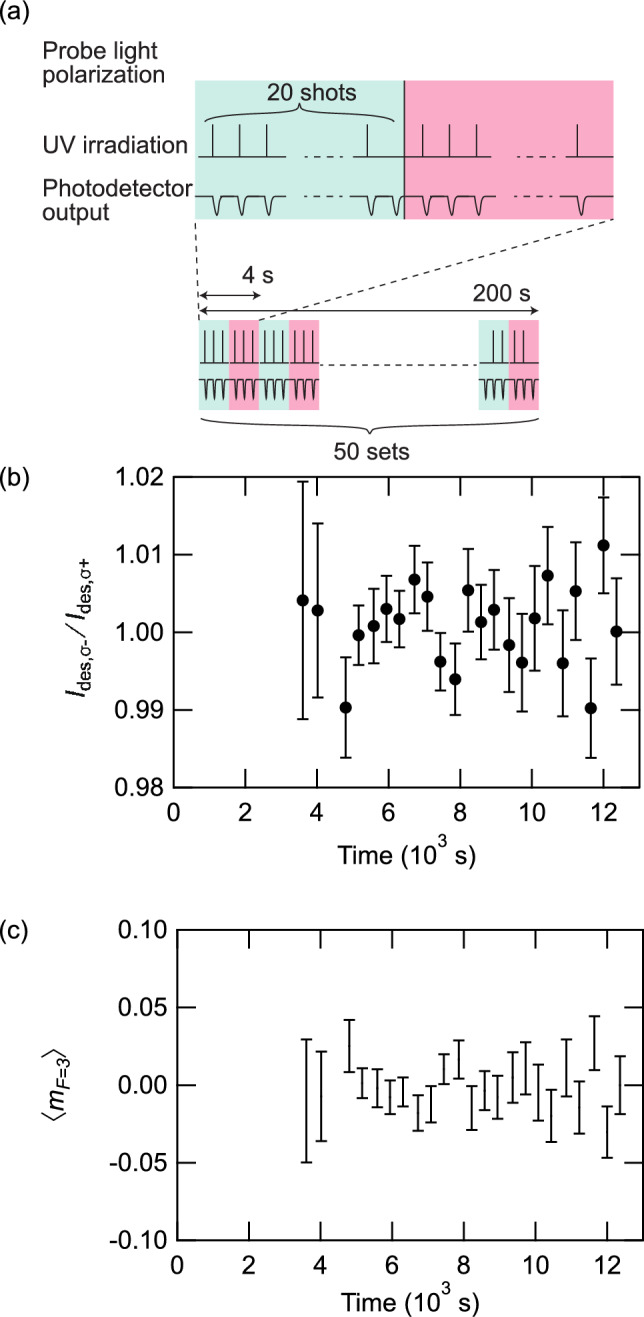
(**a**) Time sequence of the measurement of spin polarization, time evolution of (**b**) the ratio $I_{\text{des},\sigma ^-}/I_{\text{des},\sigma ^+}$, and (**c**) the averaged magnetic quantum number.

We measured the spin polarization of the desorbed atoms by obtaining time spectra of absorbance while changing the circular polarization of the probe light. Figure 5a shows a schematic diagram of the measurement procedure. Figure 5b shows the ratio $I_{\text{des},\sigma ^-}/I_{\text{des},\sigma ^+}$, where $I_{\text{des},\sigma ^-}$ and $I_{\text{des},\sigma ^+}$ are the integrated absorbance values measured with $\sigma ^-$ and $\sigma ^+$ polarized probe light, respectively. Each data point is the average of $I_{\text{des},\sigma ^-}/I_{\text{des},\sigma ^+}$ ratios calculated from 50 sets of time spectra obtained using a right($\sigma ^-$) or left ($\sigma ^+$) circularly polarized probe light. Here the quantization axis is defined as antiparallel to the probe light direction.

The ratio $I_{\text{des},\sigma ^-}/I_{\text{des},\sigma ^+}$ provides information on $m_{F=3}$, where $m_{F=3}$ is the magnetic quantum number of the desorbed atoms in the $F=3$ state. Here, we consider the transition intensities of the $F = 3 \rightarrow F' = 4$, $F = 3 \rightarrow F' = 3$, and $F = 3 \rightarrow F' = 2$ transitions of the $D_2$ line induced by the probe light. Although the probe light frequency was tuned to the $^{85}$Rb $D_2$ $F = 3 \rightarrow F' = 4$ transition line, the other two allowed transitions, which are shown by the broken arrows in Fig. 2b, were not negligible due to the Doppler shift caused by the translational movement of the desorbed atoms. At resonance, the transition probability depends on the magnetic quantum number of the ground state. For $F = 3 \rightarrow F' = 4$ transition, the transition strength $A_{3\rightarrow 4,\sigma ^{\pm }}$ for the $\sigma ^{\pm }$ transition at resonance can be written as^32^

5 $$\begin{aligned} A_{3\rightarrow 4,\sigma ^{\pm }}\propto a_{34}\left\langle m_{F=3}^2 \right\rangle \pm b_{34}\left\langle m_{F=3} \right\rangle +c_{34}. \end{aligned}$$

For $$F = 3 \rightarrow F' = 3$$, and $$F = 3 \rightarrow F' = 2$$,

6 $$\begin{aligned} A_{3\rightarrow 3,\sigma ^{\pm }}\propto a_{33}\left\langle m_{F=3}^2 \right\rangle \pm b_{33}\left\langle m_{F=3} \right\rangle +c_{33}, \end{aligned}$$

and

7 $$\begin{aligned} A_{3\rightarrow 2,\sigma ^{\pm }}\propto a_{32}\left\langle m_{F=3}^2 \right\rangle \pm b_{32}\left\langle m_{F=3} \right\rangle +c_{32}. \end{aligned}$$

The values of $a_{if}$, $b_{if}$, and $c_{if}$ are shown in Table 1.

**Table 1:** Values of $a_{if}$, $b_{if}$, and $c_{if}$.

*i*, *f*	$a_{if}$	$b_{if}$	$c_{if}$
${i}=3, {f}=4$	67.5	607.5	1350
${i}=3, {f}=3$	$-\,87.5$	$-\,87.5$	1050
${i}=3, {f}=2$	20	$-\,100$	120

The frequencies of the $F = 3 \rightarrow F' = 3$ and $F = 3 \rightarrow F' = 2$ transitions were 120.6 and 184.0 MHz lower, respectively, than that of the $F = 3 \rightarrow F' = 4$ transition^33^. $I_{\text{des},\sigma ^\pm}$ can be written as

8 $$\begin{aligned} I_{\text{des},\sigma ^\pm}\propto &\text{ }{ } d(v_{34},T) A_{3\rightarrow 4,\sigma ^{\pm }}+d(v_{33},T)A_{3\rightarrow 3,\sigma ^{\pm }}\nonumber \\{} & {} +d(v_{32},T)A_{3\rightarrow 2,\sigma ^{\pm }}, \end{aligned}$$

where $v_{if}$ is the velocity along the probe light direction of atoms undergoing $F=i \rightarrow F'=f$ transition. Here, $v_{34}$, $v_{33}$, and $v_{32}$ are 0, 94.21, and 143.7 m/s, respectively, which were calculated from the frequency differences from the $F=3\rightarrow F'=4$ transition line. $d(v_{x},T)$ is the population of desorbed atoms whose velocity in the probe-light direction is $v_{x}$, divided by $v_{z}$ to take into account that the probe light measures the density rather than the flux, which can be written as:

9 $$\begin{aligned} d(v_{x},T)\propto & {} \int \int \frac{f(\varvec{v})}{v_{z}}dv_{y}dv_{z} \nonumber \\\propto & {} \int \int \exp \left( -\frac{mv^2_{x}+mv^2_{y} +mv^2_{z}}{2k_\text{B}T}\right) dv_{y}dv_{z}\nonumber \\\propto & {} \exp \left( -\frac{mv^2_{x}}{2k_\text{B}T}\right) . \end{aligned}$$

Here, we assumed that the velocity distribution of desorbed atoms obeys the Maxwell-Boltzmann distribution and Knudsen’s cosine law. From Eq. (8), conditions $-3\le \left\langle m_{F=3}\right\rangle \le 3$, $0\le \left\langle m_{F=3}^2\right\rangle \le 9$, and $\left\langle m_{F=3}\right\rangle ^2 \le \left\langle m_{F=3}^2\right\rangle$, the temperature of desorbed atoms, and the ratio $I_{\text{des},\sigma ^-}/I_{\text{des},\sigma ^+}$, the range of values that $\left\langle m_{F=3}\right\rangle$ may take can be calculated. The range of values that $\left\langle m_{F=3}\right\rangle$ may take is indicated by the error bars in Fig. 5c. The desorbed atom temperature *T* was obtained by comparing the values of $v_{z}$ obtained by the experiment and the simulation. The results show that the $\left\langle m_{F=3}\right\rangle$ was between $-0.05$ and 0.05, a value that is at least one order of magnitude smaller than the spin quantum number of electrons ($\pm 1/2$). Because the electron spin polarization of the Fe$_3$O$_4$(001) surface at the Fermi level is $-40$–$-80$%,^20^ the present result shows that the spin polarization of the desorbed Rb atoms is significantly smaller than that of the electrons at the Fermi level of Fe$_3$O$_4$(001) surfaces. As LIAD was observed only in the multilayer regime, it is possible that the detected desorbed atoms were not in contact with the Fe$_3$O$_4$ surface, which would explain the small deviation in $\left\langle m_{F = 3}\right\rangle$ from 0. In such a case, the spin transfer to the desorbed atom is considered to be smaller than the electron spin polarization of the Fe$_3$O$_4$ surface.

**Figure 6:**
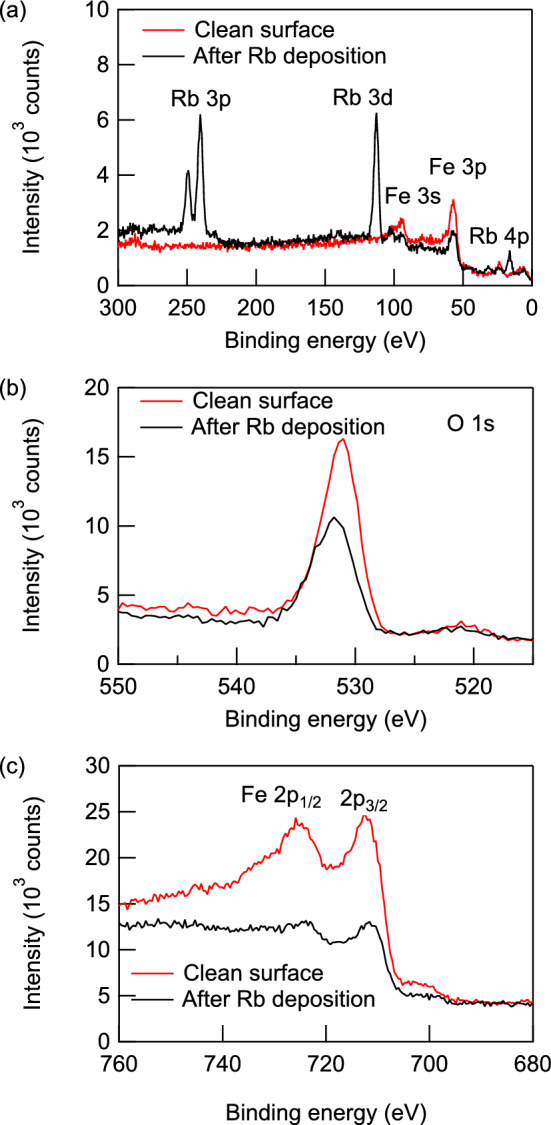
X-ray photoelectron spectra taken before and after the UV and Rb beam irradiation: (**a**) the region including Rb 3*d* and Rb 2*p* peaks, (**b**) the O1*s* peak, and (**c**) the Fe 2*p* peak.

To investigate the change in surface composition induced by UV and Rb beam irradiation, we conducted XPS measurements before and after the LIAD measurement, which included $1.26\times 10^4$ s of UV and Rb irradiation. The areas of the peaks were analyzed by subtracting the background using the Shirley method and fitting the peaks with a Gaussian function. As shown in Fig. 6a, peaks appeared at 113.1, 240.5, and 249.2 eV after the measurement, which were assigned to Rb 3*d*, Rb 2$p_{3/2}$, and Rb 2$p_{1/2}$ states, respectively. The O 1*s*-derived peak shown in Fig. 6b decreased by 30%, while the sum of Fe $2p_{1/2}$ and Fe $2p_{3/2}$-derived peaks shown in Fig. 6c decreased by 78% after measurement. The emergence of Rb-derived peaks indicate that Rb atoms were deposited on the surface. Ablation of the Fe$_{3}$O$_{4}$ film by UV irradiation is considered not to have occurred because peaks derived from the Mg atoms in the substrate, which would have been seen in the XPS spectra if ablation had occurred, were not observed. This indicates that the decrease in the O- and Fe-derived peaks was due to the deposition of the Rb atoms rather than to ablation. The difference in the decrease in the O and Fe-derived peaks may indicate that some of the oxygen diffused from the substrate to the deposited Rb layer to form an Rb oxide layer. After the irradiation, the O 2*p* peak shifted to the larger binding energy side by 0.6 eV, and the Fe $2p_{3/2}$ peak shifted to the lower binding energy side by 1.2 eV. These results indicate that the chemical bonding state of oxygen was modified and Fe atoms were reduced by Rb irradiation^34^. This supports the idea that the adsorbed Rb atoms strip oxygen atoms from Fe$_3$O$_4$ and become oxidized. We estimated the thickness of Rb oxide layer by assuming that Rb atoms existed on the surface as Rb$_2$O, which is the typical form of Rb oxide. After the measurement, the ratio $\frac{I_{\text{Rb}3d}}{I_{\text{Fe}2p}}$ was 0.49, where $I_{\text{Rb}3d}$ and $I_{\text{Fe}2p}$ are the intensities of the Rb 3*d* and Fe2*p*-derived peaks, respectively. We assumed that the intensity ratio can be written as

10 $$\begin{aligned} \frac{I_{\text{Rb}3d}}{I_{\text{Fe}2p}} =\frac{f_{\text{Rb}3d}\int _{0}^{t}\exp \left( -\frac{z}{d_{\text{Rb}3d}}\right) n_ \text{Rb}dz}{f_{\text{Fe}2p}\int _{t}^{\infty }\exp \left( -\frac{z}{d_{\text{Fe}2p}}\right) n_\text{Fe}dz}, \end{aligned}$$

where *t* is the thickness of the Rb$_{2}$O layer, $f_{\text{Rb}3d}$ and $f_{\text{Fe}2p}$ are the atomic sensitivity factors for Rb 3*d* and Fe 2*p*-derived peaks, respectively, $d_{\text{Rb}3d}$ and $d_{\text{Fe}2p}$ are the probing depth of XPS calculated for the Rb 3*d* and Fe 2*p*-derived peaks using Eq. (1), respectively, $n_\text{Rb}$ is the density of Rb atoms in a Rb$_{2}$O crystal, and $n_\text{Fe}$ is the density of Fe atoms in an Fe$_{3}$O$_{4}$ crystal. Here, $d_{\text{Rb}3d}=3.6$ nm, $d_{\text{Fe}2p}=2.7$ nm, $f_{\text{Rb}3d}=1.542$, $f_{\text{Fe}2p}$=2.952 (Ref.^28^) $n_\text{Rb}=2.58\times 10^{22}$ cm$^{-3}$ (Ref.^ 35^) and $n_\text{Fe}=3.99\times 10^{22}$ cm$^{-3}$ (Ref.^ 27^). From Eq. (10) and $\frac{I_{\text{Rb}3d}}{I_{\text{Fe}2p}}=0.49$, we obtained $t=2.3$ nm. This estimation supports the idea that LIAD occurred only in the high-coverage regime.

## Conclusions

LIAD of Rb atoms from an Fe$_3$O$_4$(001) surface was investigated using a spin-selective optical method. The coverage dependence of the desorption intensity exhibited a threshold at which the LIAD intensity began to increase. The spin polarization of the desorbed atoms was smaller than the detection limit. These indicate that LIAD of Rb from Fe$_3$O$_4$(001) occurs only in the high-coverage region and does not involve spin transfer.

## Data Availability

The datasets used or analysed during the current study are available from the corresponding author on reasonable request.
